# Analysis of clinical characteristics of mismatch repair status in colorectal cancer: a multicenter retrospective study

**DOI:** 10.1007/s00384-024-04674-z

**Published:** 2024-07-05

**Authors:** Jing Mao, Yang He, Jian Chu, Boyang Hu, Yanjun Yao, Qiang Yan, Shuwen Han

**Affiliations:** 1 Department of General Surgery, Affiliated Huzhou Hospital, Zhejiang University School of Medicine, No.1558, Sanhuan North Road, Wuxing District, Huzhou, Zhejiang 313000 People’s Republic of China; 2 Department of Oncology, The First Affiliated Hospital of Wannan Medical College, No. 92, Zheshan West Road, Jinghu District, Wuhu, Anhui 241001 People’s Republic of China; 3 Department of Gastroenterology, The Fifth Affiliated Clinical Medical College of Zhejiang, Chinese Medical University, No.1558, Sanhuan North Road, Wuxing District, Huzhou, Zhejiang 313000 People’s Republic of China; 4 Department of Oncology, Huzhou Central Hospital, Affiliated Central Hospital Huzhou University, No.1558, Sanhuan North Road, Wuxing District, Huzhou, Zhejiang 313000 People’s Republic of China; 5 Key Laboratory of Multiomics Research and Clinical Transformation of Digestive Cancer of Huzhou, No.1558, Sanhuan North Road, Wuxing District, Huzhou, Zhejiang 313000 People’s Republic of China

**Keywords:** Clinical characteristics, Microsatellite instability, Colorectal cancer, Deficient mismatch repair, Machine learning algorithm, Immunotherapy

## Abstract

**Background:**

Microsatellite instability (MSI) caused by DNA mismatch repair (MMR) deficiency is of great significance in the occurrence, diagnosis and treatment of colorectal cancer (CRC).

**Aim:**

This study aimed to analyze the relationship between mismatch repair status and clinical characteristics of CRC.

**Methods:**

The histopathological results and clinical characteristics of 2029 patients who suffered from CRC and underwent surgery at two centers from 2018 to 2020 were determined. After screening the importance of clinical characteristics through machine learning algorithms, the patients were divided into deficient mismatch repair (dMMR) and proficient mismatch repair (pMMR) groups based on the immunohistochemistry results and the clinical feature data between the two groups were observed by statistical methods.

**Results:**

The dMMR and pMMR groups had significant differences in histologic type, TNM stage, maximum tumor diameter, lymph node metastasis, differentiation grade, gross appearance, and vascular invasion. There were significant differences between the MLH1 groups in age, histologic type, TNM stage, lymph node metastasis, tumor location, and depth of invasion. The MSH2 groups were significantly different in age. The MSH6 groups had significant differences in age, histologic type, and TNM stage. There were significant differences between the PMS2 groups in lymph node metastasis and tumor location. CRC was dominated by MLH1 and PMS2 combined expression loss (41.77%). There was a positive correlation between MLH1 and MSH2 and between MSH6 and PMS2 as well.

**Conclusions:**

The proportion of mucinous adenocarcinoma, protruding type, and poor differentiation is relatively high in dMMR CRCs, but lymph node metastasis is rare. It is worth noting that the expression of MMR protein has different prognostic significance in different stages of CRC disease.

**Supplementary Information:**

The online version contains supplementary material available at 10.1007/s00384-024-04674-z.

## Introduction

Colorectal cancer (CRC) is a common gastrointestinal cancer with a high degree of malignancy and poor prognosis. According to the 2020 Global Cancer Statistics report, CRC ranks the third in incidence (9.8%) and second in mortality (9.2%) among all malignancies. The occurrence of CRC is influenced by environmental and genetic factors [1]. Among them, approximately 35% of CRC cases are related to genetic factors [2], and CRC patients with a family genetic history are mostly the result of the combined effect of gene mutation and epigenetic modification of genes [3]. The molecular pathogenesis of CRC includes three categories, namely chromosome instability, microsatellite instability (MSI), and CpG island methylation phenotype [4]. In recent years, studies have proved that MSI caused by DNA mismatch repair (MMR) defects has important significance in the occurrence, diagnosis, and treatment of CRC [5].

Microsatellites are short tandem repeat DNA sequences in the genome. The presence of new microsatellite alleles at a microsatellite site in a tumor due to the insertion or deletion of duplicate units compared with normal tissue is termed as MSI. The MMR protein maintains the genomic stability of DNA replication and reduces spontaneous mutations, including mutations in the MutS family and MutL family, among which MLH1, MSH2, MSH6, and PMS2 are the four dominant proteins. DNA mismatches that occasionally occur during replication, can be recognized and repaired by repair proteins such as MLH1, MSH2, MSH6, and PMS2. When the expression of MMR protein is lost for various reasons, including embryonic line mutation, somatic mutation, epigenetic inactivation, etc., it causes deficient mismatch repair (dMMR), which cannot detect and repair the replication errors of DNA. This results in microsatellite instability-high (MSI-H) [5, 6]; otherwise, it is called microsatellite stability or proficient mismatch repair (pMMR).

MSI-H/dMMR plays a key role in approximately 15% of CRC occurrence and development, including 3% of Lynch syndrome and 12% of sporadic CRC [7]. Lynch syndrome, an autosomal dominant genetic disease, is caused by germline mutations in the DNA MMR genes MLH1, PMS2, MSH2, and MSH6, predominantly with MLH1 and MSH2 mutations [8]. Therefore, the detection of the MMR status of CRC is of great significance to the screening of Lynch syndrome. The dMMR CRCs with lost MLH1 expression and BRAF mutation were diagnosed as sporadic MSI CRCs. In addition, MMR status can provide clinical guidance for the selection of treatment options for CRC. Studies have revealed that MSI-H/dMMR CRCs have a poor response to adjuvant chemotherapy, such as 5-fluorouracil and oxaliplatin, but have a good response to immunotherapy [9–11]. Compared with other CRCs, nivolumab provided a durable response and disease control in patients with metastatic MSI-H/dMMR CRCs [12]. Pembrolizumab had long-lasting and safe efficacy in patients with previously treated metastatic MSI-H/dMMR CRCs and was associated with significantly longer progression-free survival and fewer treatment-related adverse events than chemotherapy as a first-line treatment for metastatic MSI-H/dMMR CRCs [13]. The two immune checkpoint inhibitors have been approved by the US Food and Drug Administration (FDA) for the treatment of metastatic MSI-H/dMMR CRCs. Therefore, exploring the pathological molecular characteristics of CRC is of great significance for the individualized diagnosis, treatment, and prognosis of patients.

In this study, four MMR-related proteins in CRC tissue samples were assessed by immunohistochemistry (IHC) and 2029 CRC patients from the two centers were retrospectively analyzed to explore the relationship between MMR protein expression and the clinical characteristics of CRC. This study provides a theoretical basis for the diagnosis, treatment, and prognosis assessment of CRC.

## Methods

### Study design

This is a multicenter retrospective study of patients diagnosed with CRC. This study was approved by the Medical Ethics Committee of the hospital (No.202107002–01), and the project registration was completed in the Chinese Clinical Trial Registration Center (http://www.chictr.org.cn, No. ChiCTR2100050126). The entire study adhered to the strengthening the reporting of observational studies in epidemiology (STROBE) statement from the Equator Network (https://www.equator-network.org/).

### Patients

A retrospective analysis of the tumor pathology and clinical characteristics data of 2029 patients who underwent primary CRC surgical treatment at two centers from 2018 to 2020 was conducted. The collected data included the IHC results, sex, age, tumor location, differentiation grade, gross appearance, histological type, maximum tumor diameter, depth of invasion, TNM staging (according to the 8th edition of the AJCC CRC staging manual of CRC patients), lymph node metastasis, vascular invasion, nerve invasion, combined schistosomiasis, adenoma, etc. The included cases were all CRC patients who were diagnosed by histopathology and did not receive chemotherapy or radiotherapy before surgery. Patients with other malignant tumors were excluded. Finally, a total of 237 dMMR patients and 1792 pMMR patients were included. It is worth noting that these data can be verified in the hospital’s inpatient medical record system and routine pathological reports of patients, so there is almost no missing data. This study maintains the confidentiality of the collected patient data.

### IHC

The paraffin-embedded primary cancer tissue sections were dewaxed and hydrated, washed twice with PBS diluent (5 min/wash), soaked in 3% of H_2_O_2_ for 5–10 min at room temperature, rinsed with distilled water three times, and washed twice with PBS diluent (5 min/wash). Subsequently, 50 μl primary antibody was added dropwise to the sections, which were incubated at room temperature for 1 h or 4 °C overnight and washed with PBS diluent 3 times (2 min/wash). Biotin-labeled secondary antibody was added dropwise to the sections, which were incubated at 20–37 °C for 20 min, and then washed with PBS diluent 3 times (2 min/wash). After the final color development with DAB color developing solution, dehydration, mounting, and microscopy were performed on the sections.

The primary antibodies used for immunohistochemistry were mouse anti-human monoclonal antibody MLH1 (Clone: ES05), MSH2 (Clone: MX061), MSH6 (Clone: MX056), and rabbit anti-human monoclonal antibody PMS2 (Clone: EP51). Antibodies were purchased from Maixin Biotechnology Development, Fuzhou, China, and used according to the product instructions. The reading and grading of pathological sections were completed independently by two senior pathologists in a double-blind method, and the IHC slides with conflicting results were re-read. The judgment criteria were as follows: tumor cell nucleus with yellow or brown coloring and no background coloring was judged to be expressed, and tumor cell nucleus without yellow or brown coloring was judged to be unexpressed. If one or more of the four proteins related to MMR (MLH1, MSH2, MSH6, PMS2) were not expressed for dMMR, this indicated the presence of MSI. However, if all the above proteins were expressed for pMMR, this indicated the presence of MSS.

### Statistics analyses

Different machine learning algorithms were first used to analyze the pathological characteristics of CRC and a variety of models (logistic regression/random forest/neural network/gradient boost/catboost/support vector machine) were established. Eighty percent of the samples were divided into the training set with the use of the train_test_split machine-learning method in Python (version 0.21.2), whereas 20% were divided into the test set. The Feature_selection method was used for the selection of clinicopathological features. The accuracy and AUC value of the model were used as performance indicators to screen out the best model, and the clinical characteristics of the best model were selected for subsequent analysis. The specific method is consistent with the relevant studies published previously by the research group [14]. Afterwards, the most accurate machine learning algorithm was selected, because the clinical characteristics screening method analyzed the importance of each clinical feature in the expression of the four MMRs and statistical analysis was conducted on the most important clinical characteristics. To prevent selection bias due to confounding factors, propensity score matching (PSM) was used before statistical analyses. The ratio of 1:1 nearest neighbor matching was employed using 0.05 caliper width. The MatchIt, tableone, and foreign packages of R version 4.2.2 were used to implement PSM. SPSS 26.0 statistical software was used for data analysis. The Chi-square or Fisher’s exact probability method was used for categorical variables, the *t*-test or nonparametric test for continuous variables, and R version 4.2.2 for correlation analysis. The Corrplot package was used for Spearman rank correlation analysis, and *P* < 0.05 indicated significant difference.

## Results

### Analysis of the importance of clinicopathological features

**Fig. 1:**
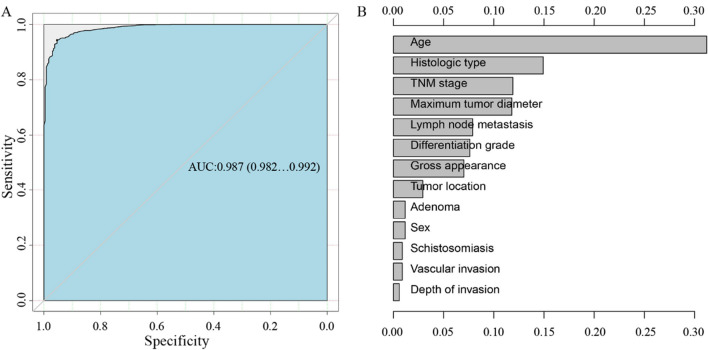
Random forest model constructed based on microsatellite instability and clinical characteristics of CRCs. (A) ROC curve of the random forest model. (B) Clinical feature screening of the random forest model. Clinical characteristics were sorted according to importance (the longer the horizontal axis is, the stronger the importance). CRC, colorectal cancer

Six machine learning algorithm models (logistic regression, random forest, neural network, gradient boosting, catboost, support vector machine) were used to train, test, and model the clinical characteristics of 2029 CRC patients. The results showed that the areas under the receiver operator characteristic (ROC) curves of the six models were 0.742, 0.987, 0.890, 0.795, 0.804, and 0.515, respectively (Supplementary Fig. 1). Therefore, the random forest model with the highest accuracy was selected to further screen the clinicopathological features, and sort them according to the importance of clinical characteristics: age, histological type, TNM stage, maximum tumor diameter, lymph node metastasis, differentiation grade, gross appearance, tumor location, whether there was adenoma, sex, whether there was a history of schistosomiasis, whether there was vascular invasion, and depth of invasion (Fig. 1).

### Comparison of clinicopathological characteristics of CRCs between the dMMR group and pMMR group

**Fig. 2:**
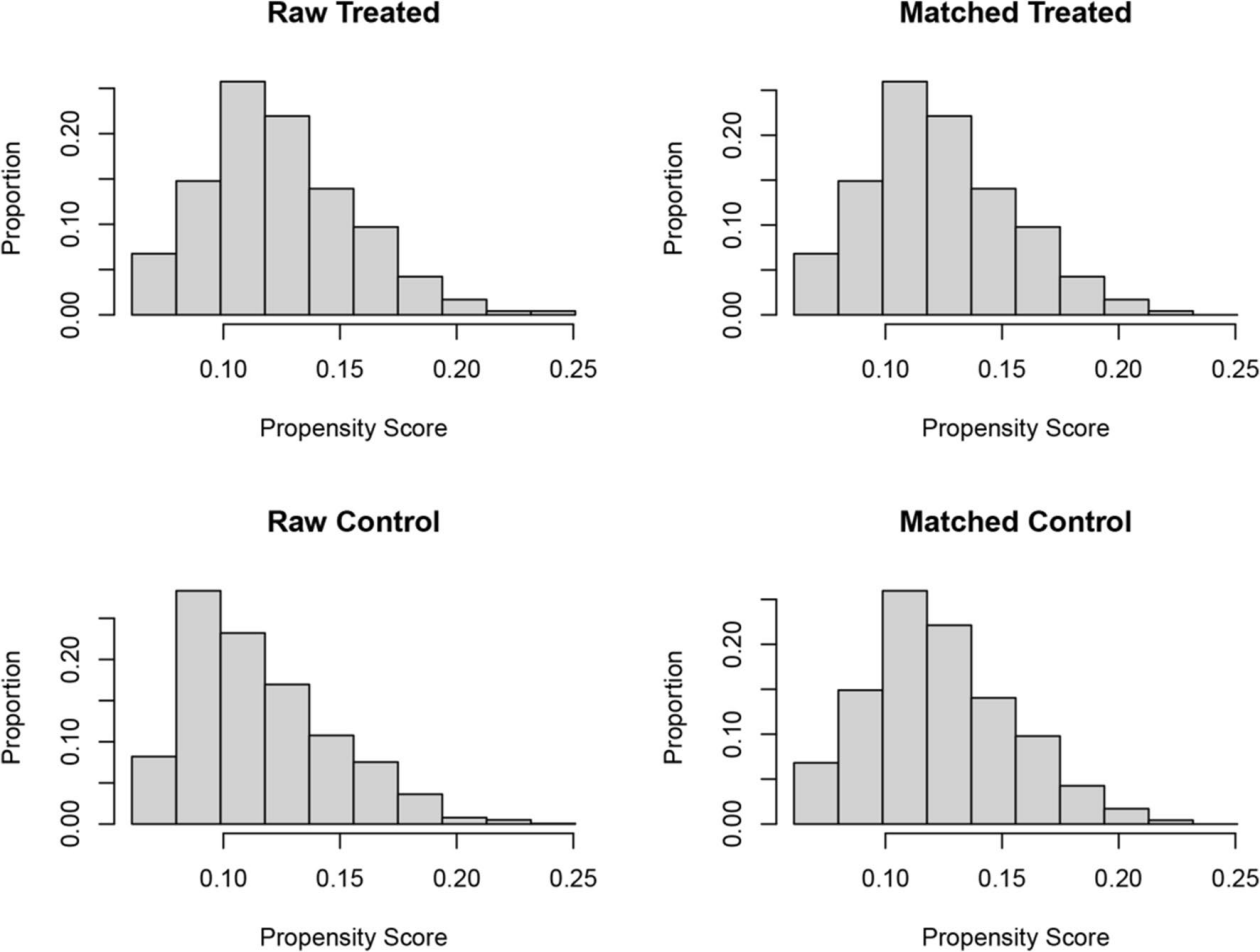
Histograms of propensity scores before and after PSM. PSM, propensity score matching

Among the 2029 CRC patients, there were 237 patients with dMMR and 1792 patients with pMMR. Before PSM, standardized mean differences (SMDs) for age and sex were greater than 0.1, which indicated poor balance between the two groups. After age and sex were included in the matching variables, 235 dMMR patients and 235 pMMR patients were paired, and SMDs were less than 0.1. In addition, histograms of propensity scores also revealed that the confounding variables were appropriately adjusted between the two groups after matching (Fig. 2). After PSM, there were statistically significant differences in histologic type, TNM stage, maximum tumor diameter, lymph node metastasis, differentiation grade, gross appearance, and vascular invasion between the two groups (*P* < 0.05) (Table 1).

**Table 1:** Comparison of clinicopathological characteristics of dMMR and pMMR CRCs before and after PSM

Group	Unmatched		*P* value	SMD	Matched		*P* value	SMD
	dMMR (*n* = 237)	pMMR (*n* = 1792)			dMMR (*n* = 235)	pMMR (*n* = 235)		
Age (years)	62.78 ± 13.31	65.08 ± 10.87	0.012	0.189	63.09 ± 12.93	63.72 ± 12.10	0.715	0.051
Sex			0.001	0.224			0.580	0.051
Male	116 (48.9%)	1076 (60.0%)			116 (49.4%)	110 (46.8%)		
Female	121 (51.1%)	716 (40.0%)			119 (50.6%)	125 (53.2%)		
Histologic type			< 0.001				< 0.001	
Adenocarcinoma	171 (72.2%)	1635 (91.2%)			169 (71.9%)	224 (95.3%)		
Mucinous adenocarcinoma	66 (27.8%)	157 (8.8%)			66 (28.1%)	11 (4.7%)		
TNM stage			0.054				0.022	
I	32 (13.5%)	251 (14.0%)			32 (13.6%)	22 (9.4%)		
II	130 (54.9%)	821 (45.8%)			128 (54.5%)	116 (49.4%)		
III	75 (31.6%)	720 (40.2%)			75 (31.9%)	97 (41.3%)		
Maximum tumor diameter (cm)	5.52 ± 2.55	4.50 ± 1.76	< 0.001		5.49 ± 2.54	4.91 ± 2.26	0.001	
Lymph node metastasis			0.001				0.007	
Yes	69 (29.1%)	720 (40.2%)			69 (29.4%)	97 (41.3%)		
No	168 (70.9%)	1072 (59.8%)			166 (70.6%)	138 (58.7%)		
Differentiation grade			< 0.001				0.003	
High	2 (0.8%)	17 (0.9%)			2 (0.9%)	0 (0.0%)		
Moderate	195 (82.3%)	1620 (90.4%)			194 (82.6%)	219 (93.2%)		
Poor	40 (16.9%)	155 (8.6%)			39 (16.6%)	16 (6.8%)		
Tumor location			< 0.001				0.076	
Left colon and rectum	128 (54.0%)	1377 (76.8%)			127 (54.0%)	146 (62.1%)		
Right colon	109 (46.0%)	415 (23.2%)			108 (46.0%)	89 (37.9%)		
Gross appearance			< 0.001				< 0.001	
Ulcerative type	103 (43.5%)	1024 (57.1%)			101 (43.0%)	181 (77.0%)		
Protruding type	124 (52.3%)	709 (39.6%)			124 (52.8%)	48 (20.4%)		
Infiltrating type	10 (4.2%)	59 (3.3%)			10 (4.3%)	6 (2.6%)		
Schistosomiasis			0.186				0.226	
Yes	10 (4.2%)	115 (6.4%)			10 (4.3%)	16 (6.8%)		
No	227 (95.8%)	1677 (93.6%)			225 (95.7%)	219 (93.2%)		
Vascular invasion			0.058				< 0.001	
Yes	34 (14.3%)	349 (19.5%)			33 (14.0%)	64 (27.2%)		
No	203 (85.7%)	1443 (80.5%)			202 (86.0%)	171 (72.8%)		
Depth of invasion			0.184				0.102	
T3/T4	200 (84.4%)	1448 (80.8%)			198 (84.3%)	210 (89.4%)		
T1/T2	37 (15.6%)	344 (19.2%)			37 (15.7%)	25 (10.6%)		
Combined adenoma			0.620				0.361	
Yes	21 (8.9%)	177(9.9%)			21 (8.9%)	27 (11.5%)		
No	216 (91.1%)	1615 (90.1%)			214 (91.1%)	208 (88.5%)		

According to the expression of MMR, CRC patients were divided into the dMMR group and pMMR group, and the clinical characteristics of each patient were collected. *P*-value was estimated using Chi-square, Fisher’s exact probability method or nonparametric test. Statistical significance, *P* < 0.05. *dMMR*, deficient mismatch repair; *pMMR*, proficient mismatch repair; *MMR*, mismatch repair; *CRC*, colorectal cancer; *PSM*, propensity score matching; *SMD*, standardized mean difference

### Comparison of clinicopathological characteristics between the MLH1, MSH2, MSH6, and PMS2 expression groups and the loss of expression group in dMMR CRCs

Among dMMR CRCs, MLH1 was missing in 161 cases, and MLH1 was expressed in 76 cases. Age, histologic type, TNM stage, lymph node metastasis, tumor location, and depth of invasion were all statistically significant differences between the two groups (*P* < 0.05). Forty cases had loss of MSH2 expression, and 197 cases had MSH2 expression. Only age showed statistical differences between the two groups (*P* < 0.05). Forty-four cases of MSH6 deletion and 193 cases of MSH6 expression were found in dMMR CRCs, and the characteristics with statistical differences, including age, histologic type, and TNM stage. There were 133 cases of loss of PMS2 expression and 104 cases of PMS2 expression among dMMR CRCs, and the differences of lymph node metastasis and tumor location were statistically significant (*P* < 0.05). The detailed information was shown in Table 2.

**Table 2:** Comparison of clinicopathological characteristics of MMR proteins expression group and loss of expression group in dMMR CRCs

Group	MLH1 ( −)	MLH1 ( +)	*P* value	MSH2 ( −)	MSH2 ( +)	*P* value	MSH6 ( −)	MSH6 ( +)	*P* value	PMS2 ( −)	PMS2 ( +)	*P* value
	*n* = 161	*n* = 76		*n* = 40	*n* = 197		*n* = 44	*n* = 193		*n* = 133	*n* = 104	
Age (years)	64.65 ± 13.12	58.80 ± 12.89	< 0.001	58.23 ± 13.47	63.70 ± 13.12	0.020	58.11 ± 13.36	63.84 ± 13.10	0.007	63.22 ± 13.99	62.21 ± 12.42	0.431
Sex			0.435			0.884			0.877			0.774
Male	76 (47.2%)	40 (52.6%)		20 (50.0%)	96 (48.7%)		22 (50.0%)	94 (48.7%)		64 (48.1%)	52 (50.0%)	
Female	85 (52.8%)	36 (47.4%)		20 (50.0%)	101 (51.3%)		22 (50.0%)	99 (51.3%)		69 (51.9%)	52 (50.0%)	
Histologic type			0.006			0.135			0.004			0.375
Adenocarcinoma	125 (77.6%)	46 (60.5%)		25 (62.5%)	146 (74.1%)		24 (54.5%)	147 (76.2%)		99 (74.4%)	72 (69.2%)	
Mucinous adenocarcinoma	36 (22.4%)	30 (39.5%)		15 (37.5%)	51 (25.9%)		20 (45.5%)	46 (23.8%)		34 (25.6%)	32 (30.8%)	
TNM stage			0.003			0.192			0.023			0.119
I	16 (9.9%)	16 (21.1%)		9 (22.5%)	23 (11.7%)		10 (22.7%)	22 (11.4%)		18 (13.5%)	14 (13.5%)	
II	86 (53.4%)	44 (57.9%)		20 (50.0%)	110 (55.8%)		25 (56.8%)	105 (54.4%)		80 (60.2%)	50 (48.1%)	
III	59 (36.6%)	16 (21.1%)		11 (27.5%)	64 (32.5%)		9 (20.5%)	66 (34.2%)		35 (26.3%)	40 (38.5%)	
Maximum tumor diameter (cm)	5.32 ± 2.45	5.94 ± 2.73	0.076	5.75 ± 3.05	5.48 ± 2.45	0.659	5.63 ± 2.22	5.50 ± 2.63	0.424	5.77 ± 2.63	5.21 ± 2.44	0.059
Lymph node metastasis			0.029			0.530			0.161			0.012
Yes	54 (33.5%)	15 (19.7%)		10 (25.0%)	59 (29.9%)		9 (20.5%)	60 (31.1%)		30 (22.6%)	39 (37.5%)	
No	107 (66.5%)	61 (80.3%)		30 (75.0%)	138 (70.1%)		35 (79.5%)	133 (68.9%)		103 (77.4%)	65 (62.5%)	
Differentiation grade			0.709			0.336			0.069			0.236
High	0 (0.0%)	2 (2.6%)		2 (5.0%)	0 (0.0%)		2 (4.5%)	0 (0.0%)		0 (0.0%)	2 (1.9%)	
Moderate	136 (84.5%)	59 (77.6%)		32 (80.0%)	163 (82.7%)		29 (65.9%)	166 (86.0%)		108 (81.2%)	87 (83.7%)	
Poor	25 (15.5%)	15 (19.7%)		6 (15.0%)	34 (17.3%)		13 (29.5%)	27 (14.0%)		25 (18.8%)	15 (14.4%)	
Tumor location			0.049			0.890			0.554			0.002
Left colon and rectum	94 (58.4%)	34 (44.7%)		22 (55.0%)	106 (53.8%)		22 (50.0%)	106 (54.9%)		60 (45.1%)	68 (65.4%)	
Right colon	67 (41.6%)	42 (55.3%)		18 (45.0%)	91 (46.2%)		22 (50.0%)	87 (45.1%)		73 (54.9%)	36 (34.6%)	
Gross appearance			0.362			0.141			0.466			0.151
Ulcerative type	69 (42.9%)	34 (44.7%)		14 (35.0%)	89 (45.2%)		16 (36.4%)	87 (45.1%)		65 (48.9%)	38 (36.5%)	
Protruding type	83 (51.6%)	41 (53.9%)		26 (65.0%)	98 (49.7%)		27 (61.4%)	97 (50.3%)		63 (47.4%)	61 (58.7%)	
Infiltrating type	9 (5.6%)	1 (1.3%)		0 (0.0%)	10 (5.1%)		1 (2.3%)	9 (4.7%)		5 (3.8%)	5 (4.8%)	
Schistosomiasis			0.839			1.000			0.347			0.718
Yes	6 (3.7%)	4 (5.3%)		4 (10.0%)	17 (8.6%)		6 (13.6%)	15 (7.8%)		11 (8.3%)	10 (9.6%)	
No	155 (96.3%)	72 (94.7%)		36 (90.0%)	180 (91.4%)		38 (86.4%)	178 (92.2%)		122 (91.7%)	94 (90.4%)	
Vascular invasion			0.052			0.390			0.532			0.731
Yes	28 (17.4%)	6 (7.9%)		4 (10.0%)	30 (15.2%)		5 (11.4%)	29 (15.0%)		20 (15.0%)	14 (13.5%)	
No	133 (82.6%)	70 (92.1%)		36 (90.0%)	167 (84.8%)		39 (88.6%)	164 (85.0%)		113 (85.0%)	90 (86.5%)	
Depth of invasion			0.019			0.073			0.057			0.783
T3/T4	142 (88.2%)	58 (76.3%)		30 (75.0%)	170 (86.3%)		33 (75.0%)	167 (86.5%)		113 (85.0%)	87 (83.7%)	
T1/T2	19 (11.8%)	18 (23.7%)		10 (25.0%)	27 (13.7%)		11 (25.0%)	26 (13.5%)		20 (15.0%)	17 (16.3%)	
Combined adenoma			0.110			0.483			1.000			1.000
Yes	11 (6.8%)	10 (13.2%)		3 (7.5)	7 (3.6%)		2 (4.5%)	8 (4.1%)		6 (4.5%)	4 (3.8%)	
No	150 (93.2%)	66 (86.8%)		37 (92.5%)	190 (96.4%)		42 (95.5%)	185 (95.9%)		127 (95.5%)	100 (96.2%)	

According to the expression of 4 MMR proteins, dMMR CRCs were divided into an expression group and a loss of expression group. *P*-value was estimated using Chi-square, Fisher’s exact probability method or nonparametric test. Statistical significance, *P* < 0.05. *dMMR*, deficient mismatch repair; *MMR*, mismatch repair; *CRC*, colorectal cancer

### The loss of MMR protein expression in CRC tissues

**Fig. 3:**
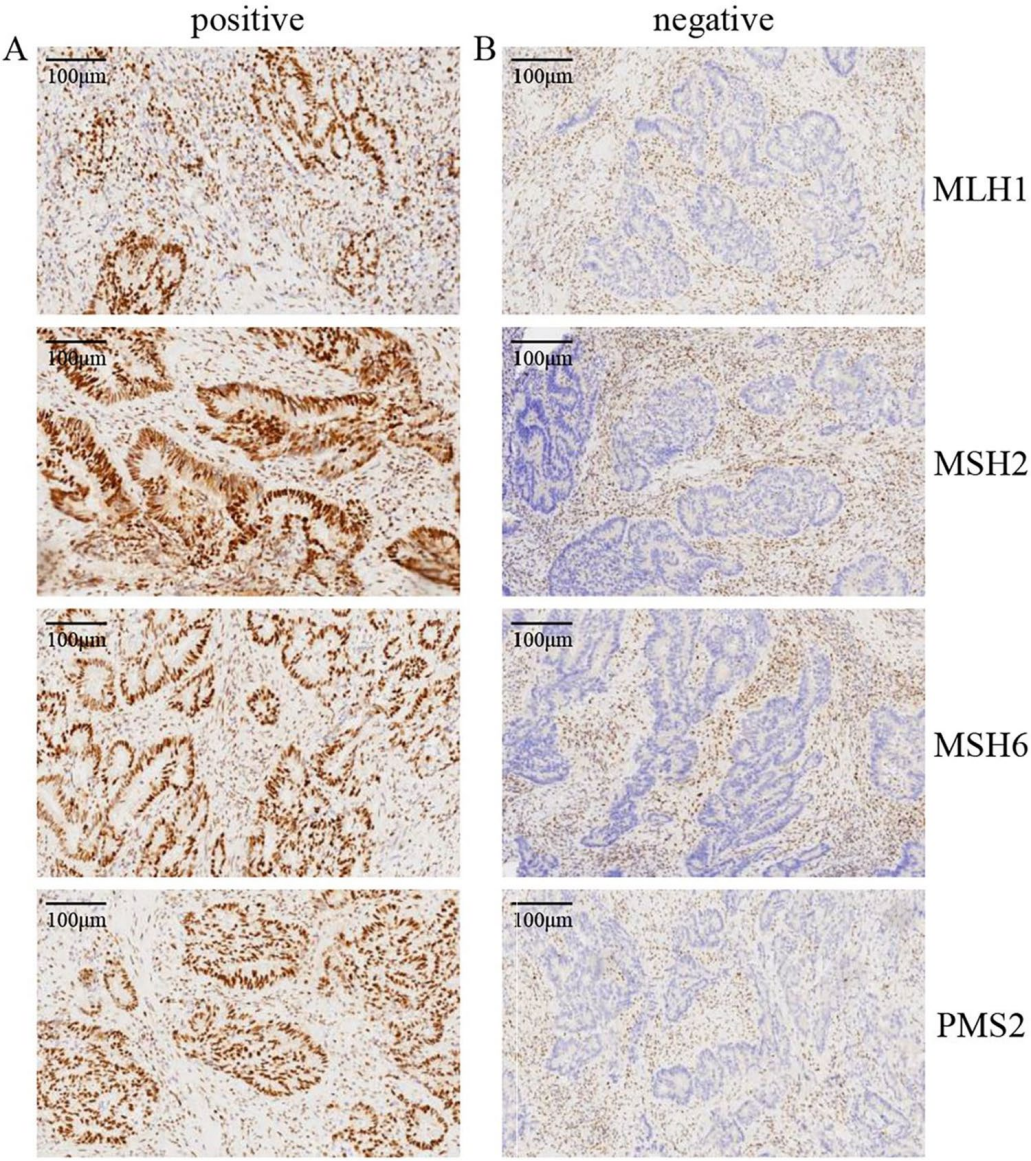
Expression and loss of MMR protein in CRC tissue (IHC, × 200). (A) MLH1, MSH2, MSH6, and PMS2 expression in CRC, respectively. (B) Loss of four MMR proteins expression in CRC, respectively. Regarding expression of MMR protein in CRC tissue, tumor cell nuclei with expression of MMR protein were stained brown, while tumor cells with loss of expression were not stained. MMR, mismatch repair; CRC, colorectal cancer

**Fig. 4:**
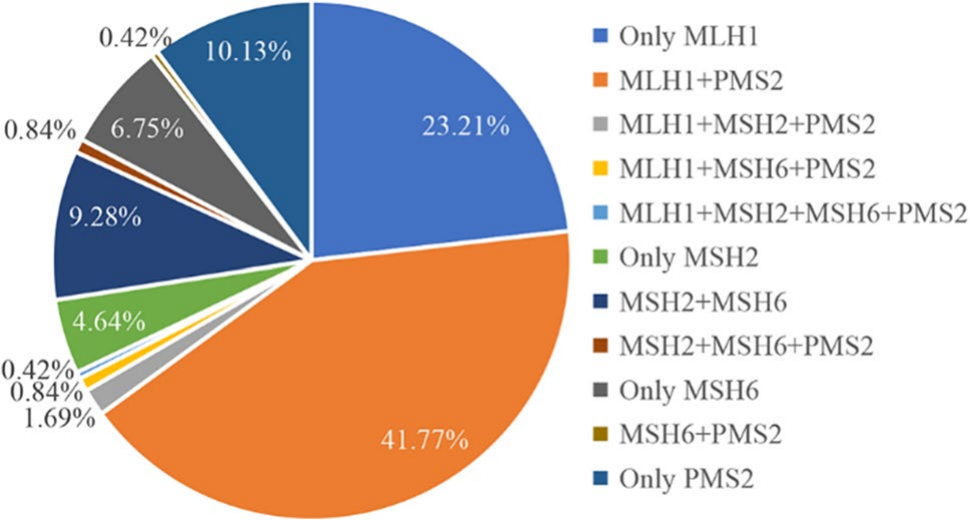
Loss of MMR protein expression in CRCs. Only MLH1/MSH2/MSH6/PMS2 meant that only one of the 4 MMR proteins was missing, and others indicated a combined absence of two or more proteins. MMR, mismatch repair; CRC, colorectal cancer

Regarding the expression of MMR protein in CRC tissue, tumor cell nuclei with the expression of MMR protein were stained brown, while tumor cells with loss of expression were not stained (Fig. 3). Among the 2029 CRC patients, there were 237 cases with missing MMR protein, accounting for 11.68% of the total. There were also 55 cases of MLH1 expression loss alone, 11 cases of MSH2 expression loss alone, 16 cases of MSH6 expression loss alone, 24 cases of PMS2 expression loss alone, 99 cases of MLH1 + PMS2 combined expression loss, 4 cases of MLH1 + MSH2 + PMS2 combined expression loss, MLH1 + MSH6 + PMS2 combined expression loss in 2 cases, MLH1 + MSH2 + MSH6 + PMS2 combined expression loss in 1 case, MSH2 + MSH6 combined expression loss in 22 cases, MSH2 + MSH6 + PMS2 combined expression loss in 2 cases, and MSH6 + PMS2 combined expression loss in 1 case (Fig. 4).

### The correlation of MLH1, MSH2, MSH6, and PMS2 expressed in dMMR CRCs

**Fig. 5:**
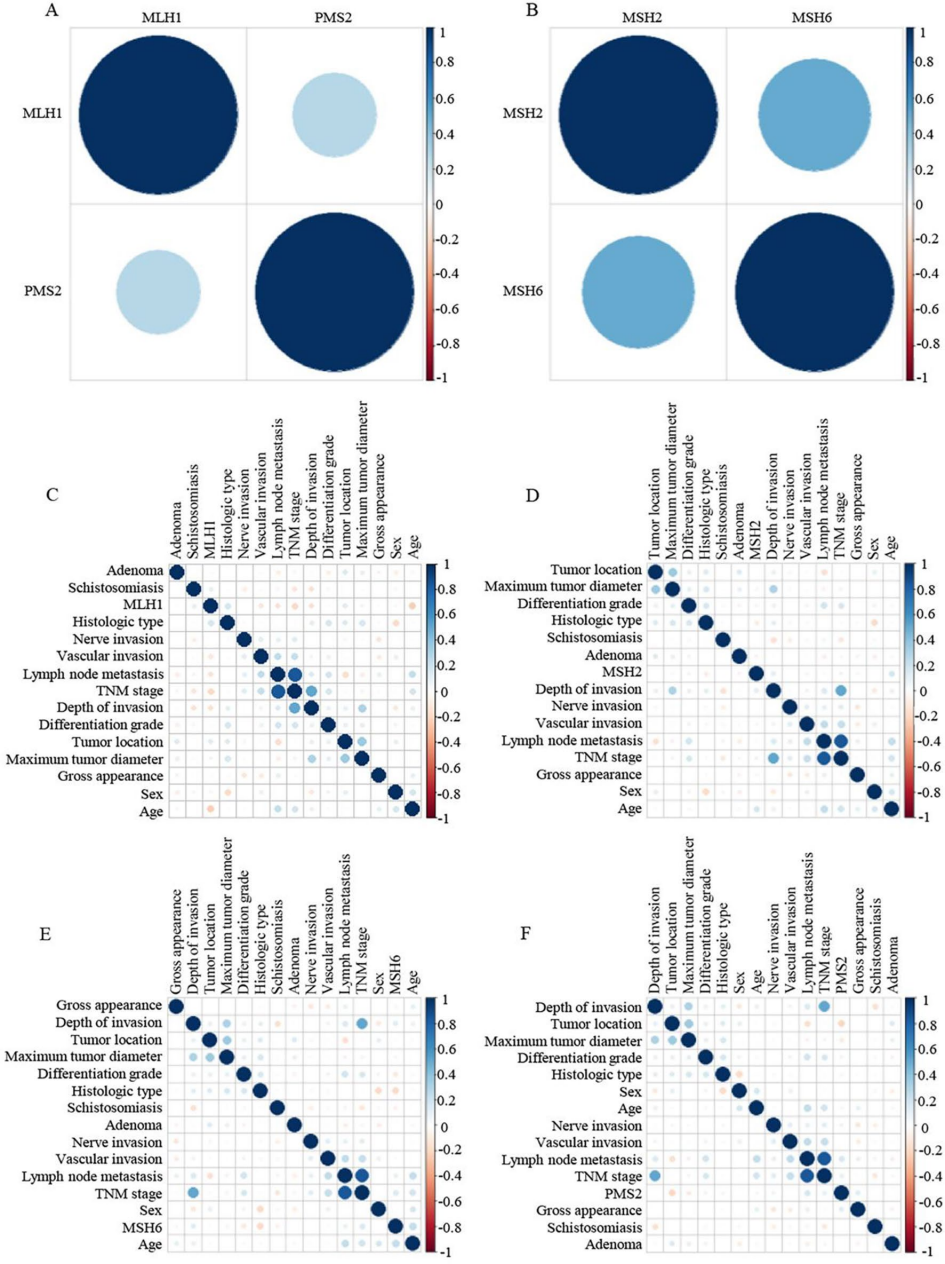
Heat map of the correlation between MLH1, MSH2, MSH6, PMS2, and clinical characteristics in dMMR CRCs. (A) Correlation between MLH1 and PMS2 (*r*_s_ = 0.285, *P* < 0.001). (B) Correlation between MSH6 and PMS2 (*r*_s_ = 0.509, *P* < 0.001). Statistical significance, *P* < 0.05. *r*_s_ represents the correlation coefficient analyzed by Spearman. When *r*_s_ > 0, it means positive correlation; when *r*_s_ < 0, it means negative correlation; when *r*_s_ = 0, it means no correlation (The larger the absolute value of *r*_s_ is, the stronger the correlation). (C) Correlation between MLH1 and clinical characteristics. (D) Correlation between MSH2 and clinical characteristics. (E) Correlation between MSH6 and clinical characteristics. (F) Correlation between PMS2 and clinical characteristics. dMMR, deficient mismatch repair; CRC, colorectal cancer

According to Spearman rank correlation analysis, the expression of MLH1 and PMS2 in 237 dMMR CRCs was positively correlated (rs = 0.285, *P* < 0.001), and the expression of MSH2 and MSH6 was also positively correlated (rs = 0.509, P < 0.001) (Fig. 5A–B, see Supplementary Table for details). The expression of MLH1 was correlated with age, TNM stage, and histological type. The expression of MSH2 had correlation with age and depth of invasion. The expression of MSH6 was correlated with age, TNM stage, and histological type. The expression of PMS2 had correlation with lymph node metastasis, gross appearance, and tumor location (Fig. 5C–F).

## Discussion

National Comprehensive Cancer Network and other guidelines believe that dMMR and MSI-H are biologically highly consistent, so it is recommended that all newly diagnosed CRC patients undergo MSI detection or MMR protein analysis [15, 16]. At present, the detection of MSI-H/dMMR is mainly carried out by polymerase chain reaction and IHC, and the sensitivity and specificity of the two detection methods can reach more than 90% [17, 18]. However, IHC has the advantages of simplicity, convenience, stability, and low price, so it is widely used for screening CRC MMR status. The relationship between MMR status and clinicopathological features in 2029 cases of CRC was retrospectively analyzed, and they were grouped according to the results of semi-quantitative analysis of IHC. Although MMR status has not been reported to be associated with schistosomiasis, some studies have shown that schistosomiasis can affect the prognosis of CRC [19]. What’s more, the patients included in this research were basically around 60 years old, and they were all from the middle and lower reaches of the Yangtze River in China. The population in this field was the high incidence of schistosomiasis in China in the last century [20]. Therefore, schistosomiasis was included as a clinicopathological feature to obtain comprehensive analysis results.

In this research study, the feature selection function of machine learning algorithms was used. The retrospective original data are often noisy. There are many independent variables that have nothing to do with the dependent variables, and only a small number of the independent variables are correlated with the outcome variables. Although it is possible to directly use conventional statistical analysis methods to perform multiple corrections to reduce errors, this will lead to a decrease in test efficiency, and machine learning algorithms can quickly and accurately screen out a small number of characteristic variables from a large number of independent variables. According to the area under the curve (AUC) of the classic algorithm prediction model, the random forest algorithm with the highest accuracy was selected. As one of the classic machine learning algorithms, the random forest algorithm can effectively analyze nonlinear, collinearity, and interactive data.

This study retrospectively collected the clinical characteristics of 2049 patients with pathologically diagnosed CRC, and the incidence of dMMR was 11.68%. Previous studies have illustrated that the loss of expression rate of MMR protein is approximately 10–20% [21, 22], which is like the results of this study. After PSM, the case data of the two groups were better comparable. Compared with the pMMR group, the proportion of mucinous adenocarcinoma, protruding type, stage I and II in the dMMR group was higher, and the average tumor diameter was larger. There was mainly moderate differentiation of tumors, but the proportion of poorly differentiated tumors was significantly higher. Lymph node metastasis and vascular invasion were less common. Therefore, patients with early dMMR CRCs have a relatively better prognosis than pMMR patients in the same period. These data are roughly like the current research results of other scholars [23, 24].

However, the proportion of mucinous adenocarcinoma and poor differentiation is relatively high in dMMR patients. At the same time, there is also evidence that the loss of MLH1 protein is closely related to tumor progression and metastasis [25]. This seems to run counter to the impression that dMMR has a better prognosis. According to the AJCC, the prognosis of right colon cancer in stages III and IV is poor, but there is no difference or better prognosis in stages I and II [26, 27]. Besides, the results of a multicenter study also showed that the prognosis of patients with MSI-H metastatic CRCs did not improve [28]. A recent meta-analysis showed that MSI-H stage I, II, and III CRCs have lower lymph node and distant metastasis rates. However, for stage IV CRCs without immunotherapy, MSI-H is comparable to any survival prognosis [22]. Another meta-analysis demonstrated that MSI-H has no effect on the prognosis of stage III CRCs, but it can increase the progression-free survival of stage IV CRCs [29]. Therefore, MSI-H can indicate a good prognosis in early CRC, while the relationship between MSI-H and prognosis in stage III and IV CRC is still controversial.

Compared with the other three proteins, inconsistent results were found in the relationship between MLH1 expression and clinicopathological features. For instance, the data for MLH1 loss shows an association with more lymph node positivity, which suggests that perhaps we should specifically consider that protein is missing when using the presence or absence of dMMR to predict the efficacy of immunotherapy in CRC patients. However, the accuracy of this conclusion still needs to be further verified by expanding the sample size or even by in vitro experiments.

In patients with dMMR CRCs, it was found that the loss of expression rate of MLH1 was equivalent to that of PMS2 (67.93% vs. 56.12%) and the loss of expression rate of MSH2 was equivalent to that of MSH6 (16.88% vs. 18.57%). The loss of MLH1 expression is often accompanied by the loss of PMS2 expression, and the loss of MSH2 expression is often accompanied by the loss of MSH6 expression. The loss of the former joint expression predominates. Further analysis proved that the expression levels of MLH1 and PMS2 were positively correlated, and the expression levels of MSH2 and MSH6 were also positively correlated, because MLH1 and PMS2 proteins and MSH2 and MSH6 proteins can form MutLα and MutSα heterodimers, respectively, and participate in the recognition and repair of mismatched DNA fragments in the form of functional protein complexes [30, 31]. Among them, MLH1 and MSH2 are the dominant proteins. When the MLH undergoes germline mutations or somatic methylation, the expression of MLH1 and PMS2 is often lost. When MSH2 has germline mutations, MSH2 and MSH6 are lost [32]. The number of simultaneous loss of four proteins expression and the combined loss of MSH2 and PMS2 expression was relatively small, with only 1 case each. False negative IHC results were not excluded, but there were similar reports [24].

The results of the correlation analysis between the 4 MMR proteins and clinical characteristics showed that prognostic indicators such as age, TNM staging, histological type, and lymph node metastasis had a significant correlation with the 4 MMR proteins, which suggested that the expression of MMR protein may have a certain relationship with prognosis.

In recent years, the application of immune checkpoint inhibitors in MSI-H/dMMR CRCs has reached a breakthrough. Nivolumab and pembrolizumab have been approved for the treatment of metastatic MSI-H/dMMR CRCs. However, regrettably, 50% of patients with advanced MSI-H/dMMR cancer will eventually progress after the immune checkpoint is suppressed [33]. This highlights the need for biomarkers that can predict the efficacy and resistance of MSI-H/dMMR CRCs to immunotherapy, so more in-depth research on the MMR protein may help address this issue.

However, this study also has some limitations. Patients with stage IV CRCs often have distant metastases and find it difficult to receive surgical treatment. Therefore, the cases included in this study lacked stage IV CRC patients, which may introduce bias. Furthermore, the survival and prognosis of CRC patients with different MMR statuses were not analyzed, and the lacked follow-up data could not show the relationship between MMR status and CRC prognosis more intuitively. We hope to address these questions in future investigations.

## Conclusions

MMR status is of great significance to the diagnosis of CRC, the choice of treatment methods, and prognostic guidance. Therefore, we chose to explore the clinical characteristics of MMR status and CRC. It was found that the proportion of mucinous adenocarcinoma, protruding type, and poor differentiation is relatively high in dMMR CRCs, but lymph node metastasis and vascular invasion are rare. It is worth noting that in different stages of CRC disease, the expression of MMR protein has different prognostic significance. Therefore, we recommend that these key populations should be tested for MMR status to receive better treatment guidance and get a better prognosis. Immunotherapy has a good effect on some dMMR CRCs, but it also has great limitations. In the future, if we want to make greater breakthroughs in this area, we must analyze the molecular mechanisms of dMMR and immunotherapy resistance in a more in-depth manner and look for potential therapeutic targets.

## Supplementary Information

Below is the link to the electronic supplementary material.

Supplementary file1 (PDF 169 kb)

## Data Availability

The datasets used and/or analyzed during the current study are available from the corresponding author on reasonable request.
