# Supplementary material for: Analysis of clinical characteristics of mismatch repair status in colorectal cancer: a multicenter retrospective study
Source: Int J Colorectal Dis. 2024 Jul 5;39(1):100. doi: 10.1007/s00384-024-04674-z (PMC11226506; doi:10.1007/s00384-024-04674-z)
Supplement: Supplementary file 1 — Supplementary file1 (PDF 169 kb) [file 384_2024_4674_MOESM1_ESM.pdf]

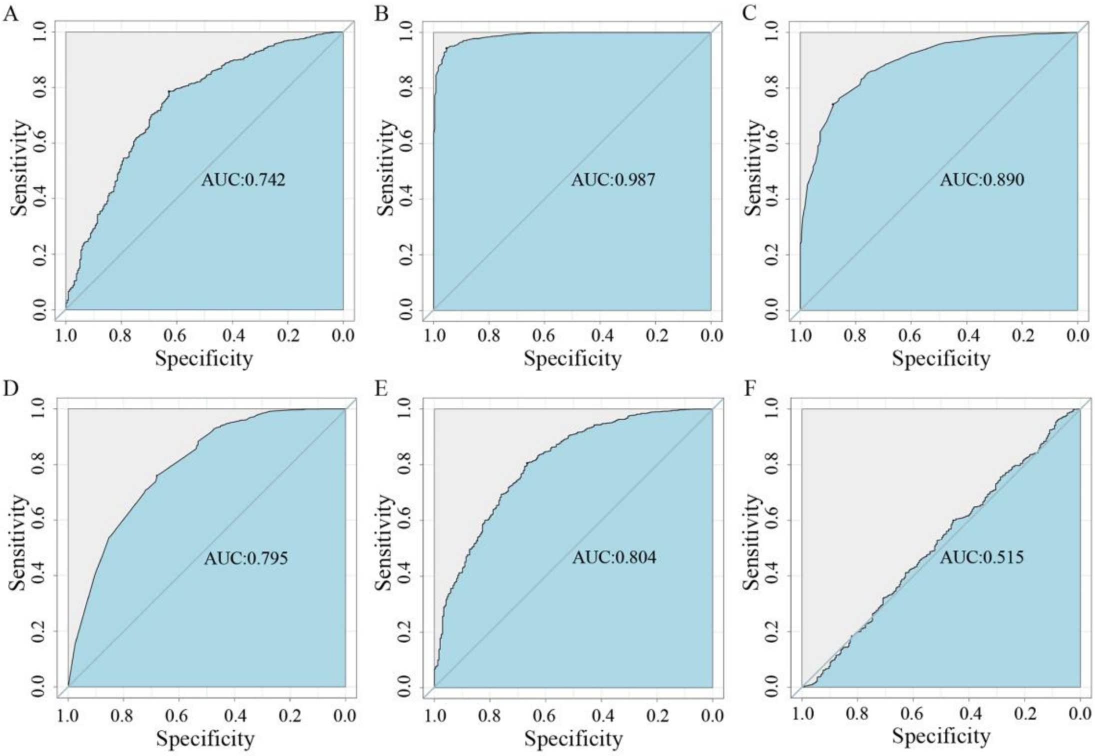

Supplementary Fig. 1 The area under the ROC curve of the six models. **A**. Logistic regression (AUC= 0.742). **B**. Random forest (AUC=0.987). **C**. Neural network (AUC=0.890). **D**. Gradient boost (AUC=0.795). **E**. Catboost (AUC=0.804). **F**. Support vector machine (AUC=0.515). The larger the AUC is, the higher the accuracy of the model is. ROC, receiver operator characteristic; AUC, area under the curve.

Supplementary Table 1 Correlation between MLH1 and PMS2 expression in dMMR CRCs.

PMS2	MLH1		r_s	P
	+	−		
+	49	55	0.285	<0.001
−	27	106		

Statistical significance, P<0.05. r_s represents the correlation coefficient analyzed by Spearman. When $r_s>0$, it means a positive correlation; when $r_s<0$, it means a negative correlation; and when $r_s=0$, it means no correlation (The larger the absolute value of r_s is, the stronger the correlation.). dMMR, deficient mismatch repair; CRC, colorectal cancer.

Supplementary Table 2 Correlation between MSH2 and MSH6 expression in dMMR CRCs.

MSH6	MSH2		r_s	*P*
	+	−		
+	178	15	0.509	<0.001
−	19	25		

Statistical significance, P<0.05. r_s represents the correlation coefficient analyzed by Spearman. When $r_s>0$, it means a positive correlation; when $r_s<0$, it means a negative correlation; and when $r_s=0$, it means no correlation (The larger the absolute value of r_s is, the stronger the correlation.). dMMR, deficient mismatch repair; CRC, colorectal cancer.
